# Isolation: The experience of adolescent motherhood in Laos

**DOI:** 10.3389/fgwh.2023.986145

**Published:** 2023-03-08

**Authors:** Souksamone Thongmixay, Dirk Essink, Taewee Kahrs, Viengnakhone Vongxay, Pamela Wright, Vanphanom Sychareun, Jacqueline E. W. Broerse

**Affiliations:** ^1^ Faculty of Public Health, University of Health Sciences, Vientiane, Laos; ^2^ Athena Institute, Faculty of Science, Vrije Universiteit van Amsterdam, Amsterdam, Netherlands; ^3^ Guelph International Health Consulting, Amsterdam, Netherlands

**Keywords:** adolescent motherhood experiences, peri-urban, exclusion, teenage pregnancy, abortion (attitudes toward)

## Abstract

**Background:**

Teenage pregnancy is a persistent public health problem with pervasive socio-economic consequences, particularly in in low- and middle-income countries, often related to low social participation and low economic security. The experiences of adolescent pregnancy and motherhood have seldom been described from a personal point of view. This study aimed to gain insights into how adolescent mothers in Laos experience their motherhood, how they perceive their situation and try to cope with it.

**Methods:**

This qualitative study was undertaken with 20 pregnant adolescents and young mothers living in peri-urban areas in two of the 18 provinces in Laos. Data were collected during 20 semi-structured interviews and two focus group discussions (*n* = 10). Digital recordings were transcribed verbatim, summarised and thematically analysed using an inductive analysis and exploratory approach.

**Results:**

The most common theme was that the young mothers experienced exclusion individually, socially and in relation to official systems. In only two cases was the pregnancy intended. All were determined to be good mothers, but were overwhelmed and unsure how to overcome structural barriers to educational, social and economic participation.

**Conclusion:**

Participants revealed that their adolescent pregnancy was tied to losses of past and future aspirations, and believed that working to prevent unintended adolescent pregnancy is worthwhile, but also advised that community support structures would help young women in their position.

## Introduction

Teenage pregnancy remains a global public health concern. Every year, an estimated 21 million girls aged 15–19 years in developing regions become pregnant and approximately 12 million of them give birth (1). Adolescents who become pregnant are more likely to be poor, with lower nutritional and health status, which highly increases the chances of maternal and child mortality and morbidity (2–5). Additionally, early motherhood may reduce options in education or employment, which will have immediate and long-term socio-economic consequences. Pregnant teenage girls often have to drop out of school, are less likely to develop valuable life skills (5), and do not have the chance to build an economic basis (4). These effects have been linked to maintaining the cyclical nature of adolescent pregnancies and poverty, and can be seen as preventing women and girls from reaching their fullest capabilities (6).

Globally, most studies into teenage pregnancy and motherhood are quantitative cross-sectional surveys about socio-ecological factors associated with teenage pregnancy, such as poor health literacy, lack of access to contraceptives and to information, and individual and societal norms that are favourable towards teenage pregnancy (7–13). The qualitative studies mainly focus on high-income countries and on pregnant adolescents, viewing pregnancy as an isolated event (8, 10, 11). Very limited research has been done into the factors underlying young people's beliefs and decisions relating the lived experience of teenaged mothers, from the onset of pregnancy through into motherhood, in low- and middle-income countries (LMICs) (11). Qualitative research can capture the unique experiences across the life-course of these adolescent mothers, their changing attitudes and behaviour, and their needs (9, 14). Such studies from high-income countries indeed indicated that being pregnant and having a baby create challenges that affect several aspects of the adolescent mother's life (15). Adolescent mothers face many physiological, psychological, social and spiritual challenges, such as lost opportunities (16), stigma, social isolation and lack of emotional support (15); they experience internal conflict between their new position as mothers and their needs as adolescents (17, 18). Additional qualitative studies in LMICs can inform us about the challenges of these adolescent mothers, their resilience, and their needs.

This research was done in Laos (Lao People's Democratic Republic), a lower-middle income country in Southeast Asia, which has undergone rapid developments during the past few years, both economic growth and changes in demographics and epidemiology, and a population transition from rural to urban areas (19). Laos has the highest adolescent birth rate in the Southeast Asian region, at 83 per 1,000 girls (20, 21). Nationally, in 2017, 33% of women were married before the age of 18, while 16.7% of girls aged 15–19 years had had a live birth or were pregnant with a first child, 1.8% of them had had a live birth before age 15, while 35% of 19-year old girls were already mothers or were pregnant (20, 21). Teenagers living in areas of low socio-economic development tend to have higher birth rates (22). Despite the high occurrence of teenage pregnancy, no (qualitative) research has been conducted into the experiences of teenagers “becoming” a mother in Laos. In particular, with the rise in urbanization, it is important to look more closely at the experience of peri-urban pregnant adolescents and young mothers, who are transitioning form rural areas where teenage motherhood is largely accepted to urban areas where it is increasingly seen as a problem (Vongxay et al.)^1^. The aim of this study is to explore how adolescent mothers in Laos experienced their motherhood and to portray their sense-making of the situation of adolescent pregnancy and their identification of future opportunities for strengthening of support structures. The results will contribute to the formulation of approaches and interventions that could improve both their health and socio-economic prospects and possibly point the way to prevention of adolescent pregnancies in future.

## Methods

### Study design

This qualitative study explored the feelings and perceptions of 20 adolescent mothers using focus group discussions (FGDs) and semi-structured interviews (SSIs).

### Study area

Data collection took place in two of the 18 provinces in Laos: Vientiane and Khammoun, chosen for their convenience as areas in the Central Region where urban development is predominant. In both Vientiane, the capital city, and in Khammoun, we collaborated with the provincial health departments to identify and contact the adolescent mothers. Vientiane has 91,575 adolescents aged 15–19 years and 26 births per 1,000 girls, while Khammoun recorded 71 births per 1,000 girls among 90,351 adolescents (23). While those coming from the rural areas in Laos are known to have higher rates of adolescent fertility and marriage under 18, two important influencing factors appear in both rural and urban areas; the teenaged mother's being in the lowest wealth quintiles, and having a low education level (24). With increasing urbanization, it is important to look more closely into the experiences of pregnant adolescents and young mothers in peri-urban areas, who have usually migrated from the rural and remote areas where adolescent pregnancy is common, accepted and mostly leads to marriage (23).

### Study population and recruitment of study participants

Adolescent mothers were eligible to participate in the current study if: (a) they gave birth to their first child between 15 and 18 years of age; (b) they gave birth to their first child in the last eighteen months; (c) the baby was still alive; (d) they were responsible for the care of their child; (e) they had resided in peri-urban Vientiane or Khammoun at least 12 months; and (f) they provided signed informed consent.

Adolescent mothers were recruited in three steps. First, the research team worked with the provincial health department to select two peri-urban districts per province. Second, they worked with the district health providers to list mothers who had given birth to their first child in hospital in the past eighteen months. Third, purposive sampling (25) was used to recruit adolescent mothers living in the study areas, through community contacts. Village health volunteers invited adolescent mothers in their community to participate in the study; if the mothers were interested, the researchers came to conduct the interviews. Adolescents who had participated in interviews were invited to join a group discussion. In both study areas, five women were willing and able to join the FGD. After analysing the interviews and conducting the group discussions, no additional themes arose; we were confident that empirical data saturation was reached in relation to our research objectives.

## Data collection and techniques

### Data collection tools

The data collection tools were structured using the Life Course Approach (26) as the basis for a mixture of descriptive and interpretive framing to explore how adolescents experienced their motherhood—keeping a focus on their lived experience, while looking into the position of each individual in their social and political environment.

**Figure 1:**
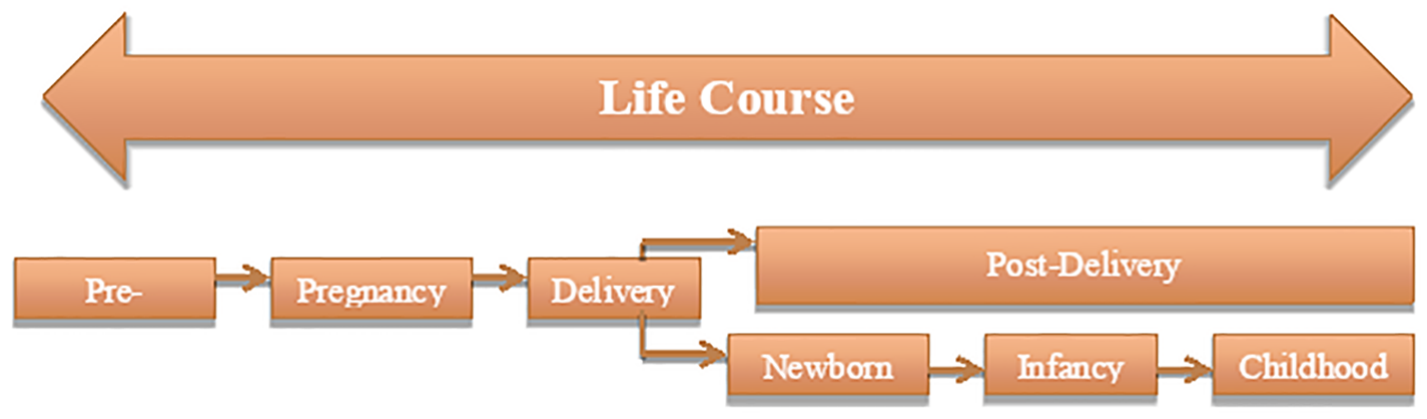
Conceptualization of life course approach to adolescent pregnancy.

Utilizing this approach places the women within a continuous timeline of each one's development, rather than isolating them as separate events as is often done in public health and bio-medical studies. Conceptualized within maternal and child social health disparity studies by Lu and Halfon (26), the life course approach posits that one's current health is the result of previous compounded experiences. It “casts health as a developmental process influenced by multiple nested social, environmental, and biological spheres that continually interact over the course of one’s life and shape the quality and nature of each person’s growth, health and development” (24). Discussions about adolescent pregnancy often focus on the nine months of pregnancy, but our approach includes the young woman's experiences from pre-pregnancy through to life with a baby (see Figure 1). Consequently, the broader environment is understood as affecting the capacity for the person to be healthy by presenting a mix of both risky and protective factors (23). The life course approach aims to identify these factors, to apply the results to creating conditions in which all mothers and children can be healthy (26).

### Data collection procedures

Given the exploratory nature of the research questions, semi-structured interviews were employed to collect data. Prior to interviews, the purpose and procedure were explained; the informed consent agreement was explained and verbal agreement secured, also for recording the interviews. The first author (a female doctor) conducted the interviews in the Lao language and steps were taken to increase comfort and decrease barriers between the participants and researcher, such as being introduced by the community contact and sitting informally. The interviews were conducted in private locations in the homes of the participants. They lasted an average of 1.5 h and were based on an interview guideline, which followed the timeline of pre-pregnancy up to the moment of interview. Open questions were asked to allow for dialogue, to uncover their experiences, decisions, challenges and needs across the timeline.

The focus group discussions utilized a guide based on the preliminary findings and themes gained from the interviews. Observational notes and audio recordings were taken during the sessions, which lasted approximately 1.5 h each.

All participants received small gifts in appreciation of their taking the time to participate.

### Data analysis

The digital recordings were transcribed verbatim, summarised and thematically analysed using an inductive analysis and exploratory approach (27). Transcripts were translated into English, then read and re-read by two researchers to identify and discuss emerging themes among the research team, to increase validity. Thereafter, all transcripts were coded. Interviews were transcribed, and their content was coded and analysed both manually and through the assistance of Atlas Ti to reveal patterns that described how the participants narrated their experiences of becoming and being an adolescent mother (27). Thematic coding was based on the key concepts of the life course approach; similar codes were categorised and clustered in sub-themes. These formed the initial coding frame, broadly related to (i) Process of becoming pregnant (ii) Pregnancy, (iii) Becoming a mother, (iv) Experience of adolescent motherhood and (v) Recommendations based on their experience of young motherhood.

### Ethical considerations

Ethical approval for the study was granted by the Ethics Committee for Health Research of the University of Health Sciences, Number 002/17, Laos, based on the WHO guideline for ethics committees (28). Legal guardians of all participants agreed to their participation by telephone and again in person before the interview. In Laos, adolescents aged 15 and above are considered able to respond to questions independently. Participants themselves were asked to confirm willingness to participate, with the knowledge that they could say no or leave the interview at any time; permission to audio-record interviews was granted by all participants.

## Results

### Social demographic characteristics

Altogether, 20 mothers participated in interviews and 10 of them also joined focus group discussions, one in Vientiane and one in Khammoun. All of the adolescent mothers were between the ages of 15–18 years, and their babies ranged from 1 to 18 months. All had left school prematurely, mostly because of their pregnancy; 16/20 girls did not progress beyond grade 9. Their partners were 18–35 years of age. One third had met their partner in the area where they were living; the others met through work, social media or at school. Most of the mothers had no previous or current work; for half of them, the main source of income was their own family. For eighteen mothers, the pregnancy under discussion was their first, and for nineteen, this was their only child. In all but two cases the pregnancy was unintended (see Table 1).

**Table 1:** Characteristics of teenaged mothers.

*P*#	Age	Grade left school	Reason left school	Age of partner	Where met partner	Work status (previous/current)	Main income source	First pregnancy	First child	Last pregnancy Intended/Unintended
1	16	8	Pregnant	18	School	None/none	Partner	Yes	Yes	Unintended
2	16	9	Pregnant	19	Digital space	None/None	Partner	Yes	Yes	Unintended
3	17	8	Finances Care of sibling	18	Area	None/None	Own Family	Yes	Yes	Unintended
4	17	11	Pregnant	25	Area	None/assist in family business	In-laws	Yes	Yes	Unintended
5	17	9	To live with partner	18	Area	None/None	In-laws	Yes	Yes	Unintended
6	17	6	Finances/To work	19	Work	Factory work/None	Partner	Second (one coerced abortion)	Yes	Intended Wanted
7	18	11	Pregnant	24	Area	None/None	Partner	Yes	Yes	Unintended
8	18	7	Finances/To work	35	Digital space	Factory work/None	Partner	Yes	Yes	Unintended
9	18	11	Pregnant	21	School	Factory work/Assist family business	Own Family	Yes	Yes	Unintended
10	18	9	Finances/To work	22	Work	Factory work/None	Partner	Yes	Yes	Unintended
11	16	5	Finance/to work	18	Area	None/None	Partner	Yes	Yes	Intended Wanted
12	17	12	Pregnant	17	School	None/None	Own family, In-laws	Yes	Yes	Unintended (Pre-term)
13	15	8	Pregnant	16	School	None/None	Own family	Yes	Yes	Unintended
14	18	9	Pregnant	1st partner 18 & 2nd partner 19	School	None/Assist mother's business	Own family	Second	First	First pregnant Unintended
15	17	9	Finance/to work	19	Area	Work in Thailand/None	Own family, Partner	Yes	Yes	Unintended
16	16	9	Pregnant	33	Beer shop	None/None	Own family	Yes	Yes	Unintended
17	17	8	Pregnant	17	Area	None/None	Own family	Yes	Yes	Unintended
18	16	8	Unruly	18	Area	None/None	Partner	Yes	Yes	Unintended
19	16	8	Pregnant	17	Beer shop	None/None	Own family	Yes	Yes	Unintended
20	16	8	Pregnant	20	School	None/None	Own family	Yes	Yes	Unintended

### Process of becoming pregnant

#### Adolescent sexual experimentation

More than two thirds of participants indicated that love for, and trust in, their partner led them to having sex. It was considered normal to have sex after four or five months in a relationship.

*“I fell in love with him, we became partners for only four months and I had sex with him.” (Participant 3, 16 years, Khammoun)*

None of participants in this study indicated that engaging in sex was under coercion in any way.

#### Lack of sexual health literacy

While two mothers said that they knew nothing at all about contraception before they got pregnant, eighteen indicated that they had some knowledge of contraception. Most said that they had some sexual and reproductive health (SRH) knowledge, for example that after a girl has a menstrual period she can get pregnant, and that contraception can prevent pregnancy, and condoms can prevent both pregnancy and Sexually transmitted infections (STIs). Fifteen adolescent mothers mentioned that they never used modern contraception, even if they knew something about it. Only five mothers indicated that they had sometimes used modern contraception (pill or emergency pill) when they had sex. The reasons given for not using contraception were: lack of ability to apply contraceptive methods, forgetting to use because of alcohol consumption, thought it was unnecessary because they did not have sex regularly, not considering it their responsibility and depending on the boy, being ashamed to buy it themselves, finding it difficult to access because there are no specific services for youth, and fearing side effects if they use contraceptives for a long time.

* “Before I became pregnant, I knew some methods that can prevent pregnancy but we just thought that we do not have sex regularly so we cannot get pregnant. Also, here there are no contraception services for youth.” (Participant 3, 16 years, Khammoun)*

#### (Un)intended

The majority of participants (18/20) indicated that their pregnancy was unintended.

*“I got pregnant last Lao New Year (April), it was unbelievable and unexpected for me.” (Participant 4, 17 years, Khammoun)*

Only two of the mothers felt a sense of readiness when facing and reflecting on their pregnancy. They explained that they had desired to have a baby early in life, especially as their partners wanted them to become pregnant as well. They felt that their pregnancy increased the family value because their families were happy with their pregnancy.

*“Yeah, I would like to have a baby. No matter the age, it is normal for girls to become pregnant one year into their marriage. My husband was happy as were my family, because we had already been married for two years.” (Participant 6, 18 years, Vientiane)*

Although some of the mothers did indicate feelings of happiness, these feelings were largely overshadowed by concerns about their and their children's current and future socio-economic situation.

### Pregnancy

#### Realization and reactions to becoming pregnant

Most of the participants did not at first recognize that they were pregnant and had not expected to become pregnant. They noticed that menstruation stopped and felt different in their bodies.

 “*I noticed that the menstruation was late and I felt extra tired, after that I consulted with my partner, who had no advice. I was still curious to know what was going on with my body, so I consulted with my close friend who has a partner. She advised me to buy a pregnancy test. I confirmed with that test.” (Participant 2, 17 years, Vientiane)*

Almost all of the adolescent mothers, only excepting the two that said they felt ready, said that they were overwhelmed and were very concerned, because the pregnancy meant that they would be unlikely to realize most of their future plans. They were aware that they could not continue their education and were unlikely to get a good job. They also feared the reactions of both their own and their partner's families. Further they worried that they would not be able to take good care of their baby.

*“I was disappointed in myself getting pregnant as a student. My family expected me to get a high level of education and a good job. I lost it.” (Participant 4, 17 years, Khammoun)*

A few adolescent mothers reported their worries that the partner and his family would not accept the pregnancy. They were particularly concerned about the mother-in-law's reaction.

*“At first when I knew I was pregnant, I didn’t know how to tell my boyfriend. I was concerned that he may not accept my pregnancy.*” *(Participant 1, 16 years, Khammoun)*

#### Disclosing and decision to continue the pregnancy

More than half of the adolescent mothers reported that they first disclosed their pregnancy to their partners. Five participants mentioned that their mothers were the first to find out and learn about their pregnancy.

 “*…Yeah, I told my boyfriend first, because he is the first person that must know about my pregnancy.” (Participant 1, 16 years, Khammoun)*

We observed different degrees of agency among pregnant girls to decide to continue the pregnancy. Three main agencies were involved in the decision about continuing the pregnancy. The first and strongest influence came from the husband's family, offering acceptance and support, the second was her own family's support, and the women's own agency came last.

##### Support from husband's family

Eight of the twenty adolescent mothers mentioned that the husband's family had the greatest influence in the decision whether or not to continue the pregnancy.

* “After my mom found out about my pregnancy, my parents met with my boyfriend*’*s parents. My parents considered abortion; it was offered as an open option by my parents if we felt we were not ready. But my husband*’*s parents supported us to continue the pregnancy and then we made the decision to continue.” (Participant 5, 17 years, Vientiane)*

Some mothers had painful memories from their partner's family about unintended pregnancy. For one participant, this was her second pregnancy but first child, because her first pregnancy was terminated by an abortion resulting from coercion by her mother-in-law.

*“I moved to my husband*’*s family after I knew I was pregnant. My mother-in-law did not really accept for me to continue with the pregnancy because there are many people in that family. She boiled some traditional herbs and gave them to me to drink, after that I felt pain in my belly and I had an abortion.” (Participant 10, 18 years, Vientiane)*

##### Support from the mother's family

After the husband's family, the family of the adolescent mother also contributed greatly to the decision to continue the pregnancy. Even if the husband's family did not accept the pregnancy, continuation was considered; they would then just compensate the girl's family. If the girl's family accepted the compensation, whether or not to continue the pregnancy was considered by her family.

*“After my mum found out, we went to discuss with my boyfriend*’*s family. They rejected my pregnancy and they paid 5 million kip (500 euro) as compensation to my family. I got support from my family to continue the pregnancy.” (Participant 3, 16 years, Khammoun)*

*“My husband and me wanted to keep the pregnancy and did not consider abortion. But my parents-in-law felt we were not ready to have a baby; they paid some money to us and wanted me to have an abortion. After that it depended on my family if they would like me to continue or to have an abortion. Anyway, my family wanted me to continue the pregnancy.” (Participant 5, 17 years, Vientiane)*

##### Their own decision

Four adolescent mothers made their own decision to continue the pregnancy: the two who had wanted to become pregnant, and two more who became pregnant unintentionally, but indicated that they had the agency to make the decision to continue the pregnancy. They did fear being forced to have an abortion.

*“I wanted to continue the pregnancy because I feared the bleeding and pain that would come with having an abortion.” (Participant 7, 17 years, Khammoun)*

Two adolescent mothers chose to keep their pregnancy secret. They tried to keep their pain, loneliness and concern to themselves, until they had an accident that led to delivery, as described for the adolescent woman whose pregnancy was revealed in hospital after a fall at home.

*“My pregnancy was secret, only my boyfriend knew. We kept it secret until my pregnancy was 27 weeks and I fell in the toilet. My mother took me to hospital and that was the first time that my family knew I was pregnant. My baby boy stayed in hospital almost two months because he was preterm.”* (Participant 4, 17 years, Khammoun)

### Visiting antenatal care (ANC)

Most of the participants indicated that they had delayed going for ANC; the first visits for ANC were usually in the second or third trimester.

“*My mum took me to ANC after she found out about my pregnancy. At that time my pregnancy was around 5 months.*” *(Participant 9, 18 years, Khammoun)*

More than half of the adolescent mothers were transferred to the Central Hospital from the district hospital; they were identified as at-risk mothers because of their age.

“*Due to my age, the doctor considered me an at-risk mother so she transferred me from Sangthong District Hospital to Mahosot Hospital.*” *(Participant 5, 17 years, Vientiane)*

Two adolescent mothers never sought ANC, because they kept their pregnancy a secret until an accident revealed it and the baby was born.

*“I never visited an ANC clinic, but I heard that pregnant women have to go for ANC. However, my pregnancy was a secret. I didn’t want anyone to know about it. Nearby my place, the ANC is only in the district hospital and it's very crowded.*” *(Participant 4, 17 years, Khammoun)*

### Experiencing adolescent motherhood

The words used to express how the adolescents experienced motherhood fall short of describing their lived experiences. In all interviews, their tears and sad faces overwhelmed the verbal narratives of hardship. Being pregnant and having a baby created challenges that affected various aspects of the adolescent mother's life. All of the mothers expressed their love for their babies, and their pride in having them and doing the mothering that women are expected to do, but most of them also talked about the challenges of caring for the baby and securing sufficient income to provide good care. The other challenge that most mentioned was being unable to complete their education due to school regulations that do not allow adolescents to continue to attend school, which would affect their future opportunities.

### Caring for the baby

This was the most challenging responsibility faced by most participants because they lacked personal experience of infant care, had to follow traditional practices after giving birth and during breastfeeding, and lacked social support.

*“…It is very difficult to take care of the baby, especially when you are young and have no job and no income to feed your baby.” (Participant 5, 17 years, Vientiane)*

“*There is no one to take care of the baby except me. […] I only have my mother to help care for the baby, but only sporadically, as my mother has to travel for work. So, the responsibility of looking after the baby is all mine.*” *(Participant 1, 16 years, Khammoun)*

*“I had to do traditional practices after birth, such as stay in hot bed, drink hot herb tea, follow food taboos, and many other things. It was very hard for me to follow these practices for three weeks. And I had to practice food taboos for almost six months after giving birth.*” *(Participant 1, 16 years, Khammoun)*

Two-thirds of the participants did not succeed in breastfeeding. Most did not have enough breast milk for the baby. A few mothers mentioned that the baby was admitted to the hospital for a few weeks because of preterm birth, so they were unable to provide breastfeeding.

“…*Exclusive breastfeeding was only for two months, because my milk was not enough for the baby and now we switched to formula milk.*” *(Participant 6, 16 years, Vientiane)*

“*My son was a preterm birth with low birth weight. He was admitted to hospital for almost three weeks. I could not provide breastfeeding for him.*” *(Participant 5, 17 years, Vientiane)*

A few of the participants mentioned that they felt a lack of attention and emotional support, and a lack of support in caring for their child, from their partner. Only their families discharged their duties and responsibilities towards the care of the child.

*“My boyfriend could not help in caring for the baby, he said that it was my duty and he had no experience in caring for a baby. Only my mum was beside me and supported me in everything.*” *(Participant 10, 18 years, Vientiane)*

Many other adolescent mothers had no help from others, which was ascribed to the fact that teenage pregnancy was frowned upon in these urbanizing communities, and support services were lacking.

“*Many people still view teen mothers as bad and think that supporting them may make other girls follow them.” (Participant 8, 15 years, Khammoun)*

*“In our place there is no social support available, such as services for young mothers where a young mother can discuss and share experiences with other mothers.*” *(Participant 5, 17 years, Khammoun)*

### Leaving school

Sixteen mothers were still in school when they became pregnant. Fear of stigmatization and school regulations barred them from continuing their education; none returned to school. While participants mentioned going back to school after delivery as technically possible, they face barriers such as stigma and fines. According to official policy, mothers must return within three months of delivery and when they do return, they must repeat the entire year during which they left. In general, the adolescents perceived a return to school as impossible due to stigmatizing behaviors and feelings of shame. As two adolescent mothers mentioned:

*“You can continue your study if no one knows you got pregnant. The school rules do not allow pregnant girls to continue to study and those who violate the school rules will be fined 500,000 kip (52 Euro).” (Participant 7, 18 years, Khammoun)*

*“Some school rules allow pregnant girls to continue their study two or three months after giving birth, but you have to stay in the same school year. Even if you passed the exam you cannot move up. But normally very few girls continue their study after delivery, because they feel ashamed to go back to school.” (Participant 6, 16 years, Vientiane)*

A few mothers mentioned that to return to school, they would have to go to a completely new school or even district to hide their motherhood.

### Loss of opportunity

Those who were forced to leave school after becoming pregnant had to adjust to the reality that their plans regarding life and career paths were lost to them. Some who were already working when they became pregnant expected discrimination if they tried to get back their jobs in the factories (all had left within three months of realizing they were pregnant). While most mothers mentioned that getting a job after delivery is not difficult because of the many factories in their peri-urban area, the needs for childcare, the working conditions in these jobs and the low pay are challenges to bio-psychosocial and financial well-being, especially of new and adolescent mothers.

*“I think if I can finish my high school, I can continue to the university and can find a good job. Or at least I can move on to study in a military school as my parents expected.” (Participant 5, 17 years, Khammoun)*

When pregnancy forces adolescent mothers to drop out of school or work, the loss is amplified by the speech and actions of peers, family and community members who chastise girls for being so irresponsible as to throw away their schooling and future opportunities, even when financial support for future education was not assured. In this study, the mothers who were close to completing secondary school had the most severe sense of loss and were most affected by accusations of irresponsibility.

*“I dreamed last night that I was still in my school uniform. I was very sad when I woke up this morning.” (Participant 10, 18 years, Vientiane)*

*“I cannot believe that I lost my dream, only five months more and I would have finished high school. I threw away my family*’*s expectations and the future opportunities in my life.” (Participant 5, 17 years, Khammoun)*

### Stigma, social isolation and lack of emotional support

Feelings of isolation as a result of informal and formal exclusion begin almost immediately after pregnancy realization for all participants. Only the two who welcomed their pregnancy were spared these feelings. Some participants began to withdraw from their families before disclosure, while all had to withdraw from school, work and social settings with peers. Furthermore, half of the participants lost contact or had reduced relationships with family or partner. These negativities created discomfort around going out in their neighborhoods or meeting and sharing experiences with new people.

*“If you got pregnant as a teenager, especially when you are studying, people in your community may blame you and your family. I feel sorry for my parents for being ashamed of my actions.” (Participant 1, 16 years, Khammoun)*

For half of the mothers, friends were reported to be the main source of stigma, and the most painful to reflect upon. The ostracization experienced upon pregnancy disclosure left the mothers both emotionally and socially isolated. In general, once girls have left school, they have no alternative spaces for cultivating close friendships; this loss was felt most strongly for girls who left school because of pregnancy. They all reported having lost considerable contact with friends, while feeling unable to make new friends.

*“Before I was pregnant I had many friends nearby. After they knew that I had an unintended pregnancy, they tried to isolate me from the group. My friends made me feel like a very bad person and tried to speak badly about me.” (Participant 5, 17 years, Khammoun)*

Even close friends are not sources of support for participants regarding their mothering experiences. Participants felt unable to discuss being a mother with friends unless they were also pregnant or a mother. Friends who were not mothers themselves would feel uncomfortable and find it inappropriate because they are all so young.

*“It is a little bit strange to talk with friends about the baby, we are still young and not experienced in how to take care of a baby. Even some of them may have experience in taking care of a baby but they are not mothers, so it is uncomfortable and inappropriate to talk with them.” (Participant 10, 18 years, Vientiane)*

Fear of stigmatization inhibited girls attempting to make new friends. Even when they know of and see other adolescent mothers in their neighborhood, social stigma and norms regarding friendships are barriers for them to initiate conversations. All mothers mentioned that they could only share personal details about their lives with a “close friend.” Close friends were those they had grown up and gone to school with; work friends turning into close friends was not considered a possibility.

*“Before I got pregnant I had many friends. But now as a young mother I have only one close friend that I can share any concerns of my pregnancy with. She never insulted me, while other friends tried to push me away because they thought I was bad.” (Participant 5, 17 years, Khammoun)*

Some girls mentioned that they still had feelings of love with their partners, but that was not enough to counter loneliness. One third of the girls discussed having no support and feeling “left out” from their own families, their partner's families and any friend groups. The other two thirds said they received support from their own family, for some also from their partner and partner's family.

*“I feel lonely. I came from another province to study here. I have only friends in school. After they knew I got pregnant, no one understands me and nobody would like to be friends with a pregnant girl of school age.” (Participant 5, 17 years, Vientiane)*

The respondents overwhelmingly felt it was their responsibility to deal with everything on their own, which was very stressful. Concurrently, while many mothers felt affected by explicit stigmatizing behavior from friends and neighbors, most said they never reacted to it. Such internalization also strengthened the mother's isolation from new relationships and potential support sources outside of their home environments.

“*Sometimes I heard they are gossiping about me, but I just didn’t respond to it, I did not take action.*” *(Participant 1, 16 years, Khammoun)*

*“My friends and neighbors talk bad things about me. I do not do anything, but I feel sad…just separate from them and just keep going. I only thought that I am just like they said.” (Participant 10, 18 years, Vientiane)*

Others gained strength from a focus on their identity as mother; their main coping strategy was derived from a sense of pride in their ability to have and take care of a child, in spite of all the hardships. They said:

*“I am proud of myself because I can do as a mother can do, like a normal mother, to take care of my baby.” (Participant 5, 17 years, Vientiane)*

The many challenges made 16 of the 20 participants feel weak, but when they think about their children they are motivated to be strong and fight for their baby to have a good life. This leads to more feelings of self-worth but does not help them socially, as their motherhood is not valued outside the home. Others focus mainly on preparing for the future of their child.

*“I only look to myself and to my baby when things are difficult and think how to help my baby become a good baby*.” *(Participant 6, 16 years, Vientiane)*

No matter the strategy, even in the absence of emotional support, the respondents felt determined to be good mothers, even though they often felt exhausted and alone in the process.

### Future life

Most of the mothers could describe future selves and experiences they could no longer access through becoming pregnant (see Table 2).

**Table 2:** Participants’ plans through the life course.

Participants (*n* = 20)	Number of Responses
**Future Plans, Pre-pregnancy**
Finish schooling (Secondary and/university)	12
Career	9
Earn money for family	8
Marriage	7
Become a mother	5
Enjoy time with friends	4
No plan	5
**Adjusted Future Plans**
Earn own money	10
Vocational training	4
Go to work abroad	5
No plan	1
**Future Plans; For Child**
Higher education	10
Good career	9
No plan	5

*“If I could go back, I would obey my parents. I didn’t want to get pregnant yet because I am not yet ready to be a mother. I have no money, no job and am not yet able to care for the baby.”* (Participant 7, 18 years, Khammoun)

Considering that nearly all the twenty pregnancies were unintended but in hindsight *wanted*, mothers described instead their wish that their pregnancies could have been *delayed* in line with their future plans. Most mothers adjusted their plans to orient them around the well-being of their baby, both immediate needs and saving money to prepare for eventual education. Upon reflection, most mothers hoped that their children would be able to fulfill aspects of the “ideal pathway” that they were unable to achieve (see Figure 1). Two mothers had planned for a university level education and three mothers hoped that their children would achieve the occupations the women had dreamed about pre-pregnancy.

“*I would like my boy to become a doctor like my dream.*” *(Participant 5, 17 years, Vientiane)*

### Recommendations based on motherhood experience

All mothers discussed the importance of using their experiences to discuss sexual relationships with their children, in hope of preventing adolescent pregnancy in their future. All planned to focus on the difficult aspects of their experience while also working to ensure their children have protected sex and/or prohibiting sexual relations in general. Even mothers who still could not articulate methods and strategies for how to best prevent an unwanted pregnancy or had not received sexual education felt that discussing sex in the future with their children would be necessary and important. And they would share the bad experience of teenage pregnancy.

*“If you want to play [have sex] you have to have safe sex.” (Participant 5, 17 years, Vientiane)*

*“I would like my child to learn about safe sex, I will try to find the way to talk in appropriate messages to her.” (Participant 7, 18 years, Khammoun)*

*“I will tell my child not to do like me, because she will lose everything in her life. To lose education, lose future plans and become a young mother is very hard and very tiring.” (Participant 5, 16 years, Khammoun)*

These plans show the adolescent mothers’ understanding that a lack of open communication and information was a factor in their own pregnancies. Yet most maintain the fear-based approach, the inherent “badness” of adolescent pregnancy, and the lack of sexual agency that they have experienced and internalized.

Four mothers stated that while they will share their difficult experiences with their child, they would not prohibit any choices or actions. They hope to create space for their child to make more informed and empowered sexual choices than they felt was possible for them, even when they themselves are still lacking information.

*“I will just support my baby and let them make their own decision.” (Participant 6, 16 years, Vientiane)*

*“Sometimes it is quite hard to prohibit a relationship, especially with teenagers, when more is forbidden, it is like more support. Just tell them how to prevent pregnancy, that*’*s fine, do not prohibit anything.” (Participant 5, 17 years, Khammoun)*

In the FGDs, each mother was asked to make recommendations for better support for pregnant adolescents and adolescent mothers (Box 1). All mothers believed it is good and worthwhile to try to **prevent pregnancies** among teenagers and to **prepare them better for motherhood** when it does happen. They suggested **specific spaces** geared towards the needs and safety of pregnant adolescents and young mothers, which could be based in hospitals/clinics or communities but never in schools.

Box 1 **Adolescent mothers' recommendations.**

• An organization that can help young mothers to access and utilize hospital-based services.
• A place where girls and women can be advised on how to prepare and deal with all things related to pregnancy, delivery and child caretaking.
• An organization to provide information in the community regarding pregnancy prevention methods as well as how to access and use them.
• Additional information after delivery for young mothers on how to take care of their babies for immediate need such as feeding the baby.
• Consultations regarding further planning, not only family planning but also practical issues like how to provide for a family after losing education or job.
• Special delivery wards in hospitals for adolescent mothers because of their special health and risk factors.
• Promotion of sexual education aimed at adolescent boys and girls so they can learn more about prevention methods, contraception.
• Organization for young mothers to come and share their experiences and advice with other adolescents, to help them prevent pregnancy until they are ready for it, either at school or outside school.
• Organization of linking someone with experience of adolescent pregnancy and motherhood to help girls who are pregnant and become mothers to deal with their worries, their fears, their plans and give general emotional support.
• Bigger campaigns to promote prevention of teenage pregnancy and increase knowledge of SRH among adolescents.
• Health care with providers who have time and are open to pregnant adolescent and young mothers to come and gain more knowledge from them.

Most participants proposed **support from peer groups**, which could include mothers of differing ages, to help adolescent mothers. All mothers desired more **information about childcare**, to decrease stress over their perceived inexperience. Moving **beyond contraception-only family planning** would help teenage mothers plan for their families in other practical ways. These recommendations closely matched the mothers' own biggest current challenges.

## Discussion

There are very few descriptions of the lived experience of adolescent mothers in the increasingly urbanizing environment in LMICs. This is the first study in Laos that explores insights into how peri-urban adolescent mothers experienced their motherhood; with particular focus on the multi-faceted exclusion though the life course stages from before pregnancy to living as a young mother. Conceptualized within maternal and child social health disparity studies, the life course approach posits that current health is the result of previous compounded experiences (26), casting health as a developmental process influenced by multiple complex social, environmental, and biological domains interacting over the course of a life, shaping the quality and nature of a person's growth, health and development (17).

It is striking, but not unexpected; to find that nearly all the twenty pregnancies were unintended and were felt by the young women to be detrimental to their educational, economic and social aspirations. Further, the girls had very little agency in deciding on whether to keep the pregnancy. The impact of the husband's family on the decision to continue the pregnancy reflects the societal structure in Laos, where women are strongly influenced by their husbands and mothers with regard to their behavior during pregnancy and lactation (29). Most of the mothers experience an internal conflict between their feelings of pride in having and caring for a baby, but also losing a future self they had aspired to. Because these young women were no longer in the rural areas where adolescent pregnancy is usually considered normal and acceptable, they faced the transitioning urban attitudes that stigmatize such pregnancies. Our respondents mainly addressed the harmful effects of teenage motherhood, and in our analysis we could not expand more on the tensions between the conflicting feelings of loss and happiness. A few additional insights on the feelings of happiness generally concerned what Vongxay et al. dubbed “traditional logic” (see text footnote 1). The girls are proud that they have become mother – what they are destined to do – and made their family proud. The young mothers also kept on emphasizing that despite their overwhelming feelings of grief, they still loved their child. But still these feelings are often overshadowed by the social and economic concerns, isolation and loss of opportunity.

Following the life course approach, initially the adolescents were unprotected because of gaps in their knowledge and their lack of ability to apply contraceptive methods. Previous studies in Laos revealed that adolescents have limited autonomous decision-making capacity, and that knowledge gaps were particularly acute for unmarried adolescents, limiting their capacity to control their reproduction, protect themselves from STIs, and utilize healthcare services (13, 30). Vongxay et al. reported that more than 65% of in-school adolescents in Laos had inadequate SRH knowledge and poor health literacy (31). A lack of knowledge and low health literacy have been demonstrated to result in less demand for contraception (32, 33). It was suggested that the youth also appreciated the importance of improving SRH literacy; they called for more SRH education, starting at a younger age. SRH education has proven to be effective in increasing not only SRH knowledge, but also utilization of modern contraceptives, and to lead to fewer adolescent pregnancies (34).

Once pregnant, the adolescent girl has limited agency in the decision to keep the child. The main agents in that decision are the families of the mother and father or other “trusted” relations. We only interviewed girls who had live births, but from their narratives it also became apparent that some girls have been advised or coerced into abortion. Such acts can have a profound influence on the girl's future physical and mental health (16). More research on this topic would be helpful in developing strategies to support the young women involved.

Adolescent mothers reported that taking care of their baby was very challenging because they lack knowledge about infant care, lack funds (their main income source is the mother's family, who may be very poor), and lack of information in general, as also found by Ngai et al. (35). However, the physical and mental wellbeing of mother and child might be improved by increasing social support, including support for her plans for the future, according to the mothers in this study and as advocated by Brown et al. (36). Many reports confirmed that social support positively influenced the mental state of adolescent mothers (17, 37–40). Young mothers believe that social support could increase their confidence in the care of their children (41). But they need that support not only from their family but also from colleagues, community and health services (17, 39).

The respondents in this study indicated that loss of emotional care, with peer stigmatization and a lack of alternative relationships, caused them the most pain. Shame and being abandoned by friends left them feeling extremely isolated. They no longer have relationships with those around them, while their pregnancy and motherhood kept them at home; they could no longer pursue their studies, work or move around in the community. They felt lonely, also in their motherhood, because most of them are the primary caregivers. The stigma related to teenage pregnancy meant most participants were ashamed and embarrassed to seek advice from other mothers, while those who had retained a few close friends felt it inappropriate to share and discuss babies with other young women who were not mothers. Most of the adolescent mothers thought that they would have experienced fewer stigmas in a rural village. Previous studies in Laos confirmed that adolescent pregnancy in rural settings is quite acceptable (13). In rural areas, if the couple can marry the pregnancy is accepted, but in the peri-urban areas, marriage was mainly a way to protect the young woman from worse forms of stigmatization, including exclusion from sexual and reproductive healthcare, including access to contraceptives (42).

A previous study in Laos indicated that being closer to the city gave many adolescents, and their families, higher expectations for education and careers, even though these expectations did not always match with the financial reality of their families (23). Similarly, we found that the higher expectations among our per-urban respondents also resulted in greater disappointment when pregnancy interfered with their plans. As a result, most mothers experience internal conflicts about the loss of their desired future as a result of the inadvertent acquisition of a child, but still want and love the child. This reaction is common, because adolescent mothers are often undermined by feelings of loss, especially the inability to graduate from school and get a good job, along with the feelings of loss and regret associated with past life and expectations for the future (17, 40). Studies in Thailand also found that the core theme amongst adolescent mothers was “living with conflict between needs as a mother and as an adolescent” when all participants had reported being unprepared to become a mother (43).

Counter to mainstream literature, recent studies are also finding that becoming a mother can actually increase an adolescent's sense of purpose, responsibility, maturity and perseverance in education (44). Ambitions for life goals, adjusted after pregnancy realization, have been found to increase (6, 17, 45), which most mothers in this study also exhibited. Studies highlighting the positive sense of self and belonging through adolescent motherhood are generally from the U.S., U.K. or Australia, where there are more structural protections and opportunities for pregnant adolescents and mothers (46–48).

### Strengths and limitations

The results of this study are probably generalizable to other peri-urban areas in Laos with adolescent mothers who had their children, because results in Vientiane and Khammoun were comparable. Considering that the scarce data in the literature reveal similar findings, it is likely to reflect the situation in peri-urban settings of countries in transition. We have no information about those who did not continue their pregnancies. The interviews were difficult and lengthy because the young women were in pain recalling their difficult times and often in tears; those feelings cannot easily be reflected in the text. Additionally, we interviewed the young women but not their families, so we have their point of view but may have missed information relevant to their future support. The FGDs increased internal validity through triangulation as well as provided recommendations from the perspective of young motherhood. Parental and guardian consent was not required by the Committee because in Laos minors, considered to be adolescents 15 to 18, can give con- sent themselves. Parental consent would be needed for children under 15 years of age. There are 4 adolescent mothers who were 15, others were 16 or 17 under another guardian can confirmed their consent to participate the study.

This study was one in a series about adolescent pregnancy carried out in the course of a PhD study; previous studies with a more quantitative approach led to the realization that a qualitative study was also needed. The main researcher is a female medical doctor with whom the young women apparently felt safe to confide personal information, but may have influenced their focus on the problems more than the pleasures in their life. The researchers considered major challenges that adolescent mothers faced, and what reflexivity brings to the forefront. We have an experienced research supervisor, arranging pilot interviews that include active feedback on interviewing style from interviewees, and being reflexive during interviews.

## Conclusion and recommendations

This study describes the lived experienced of adolescent mothers in Laos, who experienced their transition to and through motherhood as dominated by diverse types of exclusion and a lack of agency. The participants reflected on adolescent pregnancy as tied to losses of past and future aspirations, and believed that working to prevent unintended adolescent pregnancies is worthwhile. They were proud of their motherhood status. Adolescent mothers need more social support to ensure that they are better prepared for future pregnancies. Ultimately, despite their stigmatization, participants feel that they are actors who can engage both informally with other girls and mothers as well as formally with health providers and organizations, which can help to decrease the pain of exclusion for future mothers.

## Data Availability

The original contributions presented in the study are included in the article/Supplementary Material, further inquiries can be directed to the corresponding author.

## Footnotes

^1^Vongxay V, Thongmixay S, Steenbergen E, Sychareun V, Broerse JEW, and Essink DR. Contrasting perspectives of adolescents and other stakeholders on causes of teenage pregnancy in Lao PDR: why is it still high? (2020, in review).
